# RETRACTION: Effects of Application of the Clinical Nursing Pathway on Surgical Site Wound Infection and Postoperative Complication Rates in Patients with Total Knee Arthroplasty: A Meta‐Analysis

**DOI:** 10.1111/iwj.70168

**Published:** 2024-12-15

## Abstract

**RETRACTION:** HanJ. and LiuX.‐J. , “Effects of Application of the Clinical Nursing Pathway on Surgical Site Wound Infection and Postoperative Complication Rates in Patients with Total Knee Arthroplasty: A Meta‐Analysis,” International Wound Journal 21, no. 2 (2024): e14469, 10.1111/iwj.14469.PMC1082852337890862

The above article, published online on 27 October 2023, in Wiley Online Library (http://onlinelibrary.wiley.com/), has been retracted by agreement between the journal Editor in Chief, Professor Keith Harding; and John Wiley & Sons Ltd. Following an investigation by the publisher, all parties have concluded that this article was accepted solely on the basis of a compromised peer review process. The editors have therefore decided to retract the article. The authors did not respond to our notice regarding the retraction.

**RETRACTION:** J. Han and X.‐J. Liu , “Effects of Application of the Clinical Nursing Pathway on Surgical Site Wound Infection and Postoperative Complication Rates in Patients with Total Knee Arthroplasty: A Meta‐Analysis,” International Wound Journal 21, no. 2 (2024): e14469, 10.1111/iwj.14469.PMC1082852337890862

The above article, published online on 27 October 2023, in Wiley Online Library (http://onlinelibrary.wiley.com/), has been retracted by agreement between the journal Editor in Chief, Professor Keith Harding; and John Wiley & Sons Ltd. Following an investigation by the publisher, all parties have concluded that this article was accepted solely on the basis of a compromised peer review process. The editors have therefore decided to retract the article. The authors did not respond to our notice regarding the retraction.

